# Sacrospinous hysteropexy with a low weight transvaginal polypropylene mesh for treatment of complete uterovaginal eversion

**DOI:** 10.1590/S1677-5538.IBJU.2018.0555

**Published:** 2019-09-02

**Authors:** Arnold P. P. Achermann, Éder S Brazão, Cássio L. Z. Riccetto, Paulo C. R. Palma

**Affiliations:** 1 Divisão de Urologia Feminina, Departamento de Cirurgia, Faculdade de Ciências Médicas, Universidade de Campinas, UNICAMP, Brasil

## Abstract

**Introduction:**

Pelvic Organ Prolapse (POP) is a common condition in elderly resulting from the weakening of the organ suspension elements of multifactorial origin. It compromises significantly the quality of life and can affect more than 50% of multiparous women. Stage IV prolapse or complete uterovaginal eversion corresponds to 10% of the cases and the only form of curative treatment is the surgical correction. The aim of this video is to demonstrate our technique of sacrospinous hysteropexy with a low weight transvaginal polypropylene mesh for treatment of this challenge condition, focusing on technical details in order to prevent mesh related complications. Major, but rare complications, include: infection, prolapse recurrence, abscess formation, bladder perforation and urinary fistula. These situations are related mostly to low volume centers.

**Materials and Methods:**

A 70 years old female with a stage IV POP had obstructive lower urinary tract symptoms. Only after reducing prolapse, it was possible to urinate, but without stress urinary incontinence. No topic estrogen was prescribed before the surgery and she also didn´t take any kind of hormone replacement therapy. Transvaginal ultrasound and the Pap smear screening were done with normal results. Cystoscopy wasn´t employed at anytime of this procedure. Hydrodissection of vaginal wall was followed by longitudinal incision from the level of bladder neck to the cervix. Notice that the ideal dissection should maintain the vaginal thickness, and address the plane of the connective tissue between the bladder and the vagina. Bladder base is then released from the anterior aspect of the cervix in order to create a site to pericervical ring repair and to fix the apex of the Calistar Soft® with polypropylene 3.0 stitches. A blunt dissection extended downwards through the lateral aspect of the *levator ani* fascia till the identification of the ischial spine and sacrospinous ligaments bilaterally. Two polypropilene 2.0 threads mounted on a specially designed tissue anchor system (TAS) are then fixed into each sacrospinous ligament 1.5 to 2 cm away from the ischial spine and repaired for further prosthesis anchoring. Then, a longitudinal incision is done at the posterior vaginal wall and the recto-vaginal fascia detachment from the posterior aspect of the pericervical ring is identified and corrected with interrupted polypropylene 2.0 stitches to the cervix and to the pericervical aspect of elongated uterosacrus ligaments bilaterally. The Calistar Soft A (anterior) and P (posterior)® prosthesis were fixed at the anterior and posterior aspects of the cervix, respectively, with interrupted polypropylene 3.0 stitches and meshes’ arms are fixed to the sacrospinous ligament using the previously implanted TAS. Then, the distal Calistar Soft A® arms were bilaterally fixed into the internal obturator muscles using its fish spine–like multipoint fix device in order to prevent mesh folding. Finally, perineal body repair was done and vaginal wall was closed with individual absorbable interrupted polyglactin 2.0 sutures and a 16 Fr Foley catheter as well as a vaginal pack embedded on neomicin-bacitracin cream were kept overnight.

**Results:**

A high satisfaction rate has been computed with synthetic mesh to POP surgery correction. Approximately 10% of cases of mesh exposure may occur, most of them oligosymptomatic and easy handed by excision or with topic estrogen preparations. After 1 year follow-up, our patient is still satisfied without any complain and no relapse. Conclusion: We described a successful treatment of stage IV POP in an old female patient. This technique can be used for advanced end stage POP patients, especially those with some contraindication to sacropromontopexy, but who want to keep vaginal length and uterus. Anatomical knowledge, obedience to technical care, and intensive training are the keys for minimizing the risk of complications. Although we had success with this technique, more studies with proper randomization are necessary to compare success and complications of sacrospinous hysteropexy with a low weight transvaginal polypropylene mesh to sacropromontopexy.

**ARTICLE INFO**

*Arnold P. P. Achermann*

http://orcid.org/0000-0002-6736-2060

Available at:  http://www.intbrazjurol.com.br/video-section/20180555_Achermann_et_al

Int Braz J Urol. 2019; 45 (Video #16): 856-7

